# The Comparative Evaluation of the Morphology and Dimensions of the Sella Turcica in Skeletal Class III Patients and Patients With Unilateral Cleft Lip and Palate in Post-Pubertal Age Group

**DOI:** 10.7759/cureus.29730

**Published:** 2022-09-29

**Authors:** Sumukh Nerurkar, Ranjit Kamble, Sunita Shrivastav, Abhishek Sanchla, Japneet Kaiser, Jeni Mathew, Nandalal Toshniwal

**Affiliations:** 1 Department of Orthodontics, Sharad Pawar Dental College, Datta Meghe Institute of Medical Sciences, Wardha, IND; 2 Department of Orthodontics, Rural Dental College, Pravara Institute of Medical Sciences, loni, IND

**Keywords:** cleft lip and palate, sella turcica bridging, dimension of sella, lateral cephalogram, sella turcica

## Abstract

Background

The clinical features of unilateral cleft patients have great similarity to class III patients, viz., ANB <2° and Wits appraisal <-3 mm. In this study, we determined the frequency of various shapes of the sella in cleft and class III patients. We also measured the dimensions of the sella turcica in the aforementioned groups. Studying the morphology and dimensions of the sella in different groups can help us to understand the role of the cranial base in the development of malocclusion.

Material and methods

The study is composed of 46 patients, divided into two groups with an age range of 14 to 21 years. The linear dimensions of the sella were measured, and the shape of the sella was determined. Comparison was done between the two groups using Student’s t-test.

Result

When the two groups were compared, length was found to be similar in both groups, but depth and diameter were greater in the class III group. An abnormal shape of the sella was found predominantly in both class III and cleft cases. The normal morphology accounted for only 13.04% of all the cleft patients, whereas 39.13% of the class III cases exhibited a normal shape of the sella. Sella turcica bridging was seen in 30.43% of cleft patients as opposed to 21.73% of class III patients exhibiting the bridging.

Conclusion

With the finding that 74% of the subjects exhibited abnormal morphology of the sella, confirming that it can be one of the factors for malocclusion must be given more consideration.

## Introduction

The sella turcica of the sphenoid bone is a depression on the superior surface of the skull's base [1]. The sella turcica resembles a saddle used by the Turkish and thus owes its name to two Latin words, that is, sella that means a saddle and turcica that means Turkish [2]. It is present in the middle of the sphenoid bone, just behind and above the sphenoid sinus. It is medial to the cavernous sinus, anterior to the pontine cistern, and inferior to the diaphragm sella. The depression present in the saddle is known as the hypophyseal fossa, which houses the pituitary gland. The sella turcica not only protects but also stops the pituitary gland from overexpanding [3]. The sella turcica area plays a crucial role during embryological development. Specifically, it mediates the migration of the neural crest cells to the maxillary and frontonasal developmental fields.

There have been many landmarks that help the orthodontist to analyze the lateral cephalogram and in turn help in planning the treatment [4]. One such landmark is the sella turcica. This landmark is located in the craniofacial region, and it assists the orthodontist to calculate the orientation of the mandible and the maxilla in relation to the cranial base [5].

Among the congenital anomalies, cleft lip and palate represent the second most common orofacial malformation in live births. In the Indian subcontinent, out of a total of 24.5 million births per year, cleft lip and palate consist of 27000-33000 per year [6]. Patients suffering from cleft lip and palate display differences in craniofacial morphology when compared to non-cleft class I patients [7]. These differences include concave profile and anterior and posterior crossbite, which is evident clinically, and a shorter maxilla with retrusion, clockwise rotation of the mandible, increased gonial angle, increase in the anterior facial height, reduced posterior facial height, and an increased mandibular plane angle, which can be confirmed on the lateral cephalogram [8].

It can be noted that the skeletal features of the patients with unilateral cleft have a high resemblance to those in patients with skeletal class III pattern. So, we have conducted this study with the aim of providing normative reference data about the dimensions and morphology of the sella turcica and due to the paucity of studies that provide a comparison of these measurements of the sella turcica in patients with unilateral cleft lip and palate and patients with skeletal class III pattern.

## Materials and methods

Subjects and methods

Study Population

**Figure 1:**
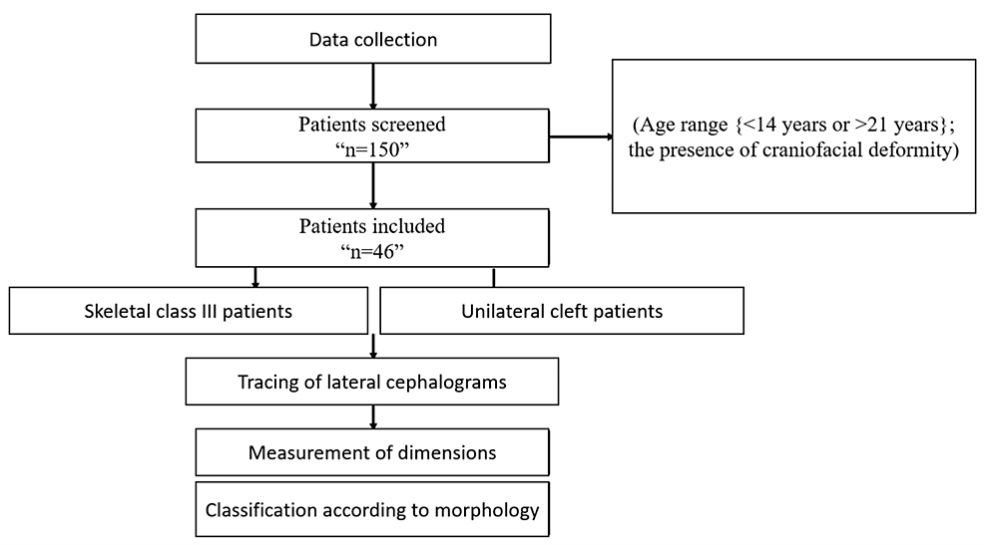
Graphical representation of the method followed This depicts the graphical method used for the study

The data was acquired from lateral cephalometric radiographs from Sharad Pawar Dental College Archive, Wardha. Lateral cephalogram is regularly used in orthodontics. These radiographs have now become indispensable to orthodontists in deciding the treatment plan of a particular patient. This retrospective study included 46 patients. All the procedures were done after receiving approval from the Institutional Ethical Committee of Datta Meghe Institute of Medical Sciences (DMIMS) (reference number DMIMS (DU)/IEC/2021/360). The patients were categorized into two groups: unilateral cleft patients and non-cleft patients with skeletal class III patterns. The patients were screened by an operator for the following inclusion criteria: having age between 14 and 21 years, the presence of unilateral cleft, or non-cleft patients with skeletal class III pattern (ANB <2° and Wits appraisal <-3 mm) [9]. Patients with any kind of craniofacial syndrome were excluded from the study (Figure 1).

The sample size calculation (SPSS version 27.0 {IBM SPSS Statistics for Windows, Armonk, NY} and GraphPad Prism version 7.0 {GraphPad Software, San Diego, CA}) performed for Student’s t-test indicated that the total sample size needed was 45 subjects. The power of 80% was considered, and the probability of a type I error was 0.05. A total of 150 patients were screened using the eligibility criteria. One hundred four out of these 150 subjects were excluded from the study based on not meeting the inclusion criteria. Therefore, the study was composed of 46 patients [10]. Lateral cephalograms of the 46 patients were gathered and separated into two groups. Anonymization of the radiographs was done by assigning each with a unique numeric code.

Cephalometric Tracing

A thin acetate paper with thickness of 0.003 inches was used by the author to trace the sella turcica on all the lateral cephalograms [11]. Optical illumination was used to aid the tracing. A digital vernier caliper was then used for measuring the dimensions of the sella. The following structures were traced: tuberculum sella, floor and dorsum sella, anterior clinoid process, and posterior clinoid process [1].

Measurement of Size

**Figure 2:**
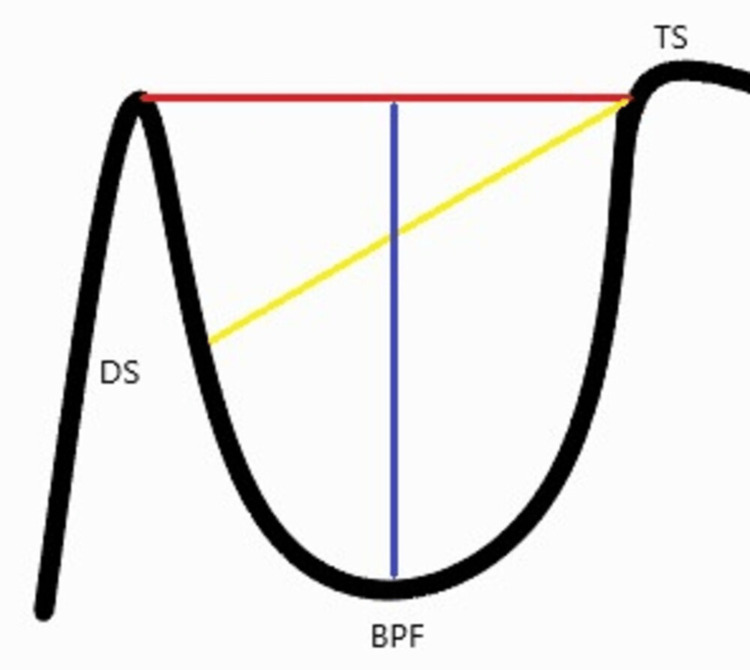
Dimensions of the sella turcica TS: tuberculum sellae; BPF: base of the pituitary fossa; DS: dorsum sellae The red line depicts the length of the sella. The blue line depicts the depth of the sella. The yellow line depicts the anteroposterior diameter

All the reference lines required for the measurements were in the median plane. The length was calculated by measuring the distance between the dorsum tip of the sellae and the tuberculum sellae [12]. The depth was calculated as the perpendicular distance between the aforementioned line and the deepest point on the floor. The diameter was measured from the tuberculum sellae to the farthest point on the fossa’s posterior wall (Figure 2) [13].

Assessing the Morphology

The morphological assessment of the sella was done in accordance with the already existing morphological types given by Axelsson et al. [9]. According to them, there are five morphological variations present apart from normal. They are as follows: oblique anterior wall, double contour of the floor, sella turcica bridge, irregular notching in the posterior wall of dorsum sellae, and pyramidal shape of dorsum sellae [9]. The morphology assessment also involved checking for the presence of a bridge, which is a variation in the morphology characterized by excessive ossification of the ligaments between anterior and posterior clinoid process.

## Results

Size of the sella turcica

When a comparison of linear dimensions was done between the cleft group and the class III group, a statistically significant difference was found in the depth (p<0.02) and diameter (p<0.03), whereas no significant difference was obtained when comparing the length (p>0.11). The depth and diameter of the sella were greater in the cleft group. It was also found that the depth and diameter of sella are considerably greater in the cleft group when compared to the class III group (Table 1).

**Table 1:** Comparison of linear dimensions of the sella U/L: unilateral cleft lip and palate; N: number of patients; SD: standard deviation

		N	Mean	SD	Minimum	Maximum	P-value
Length	U/L cleft	23	8.287 mm	1.411	6.1 mm	11 mm	-
	Class III	23	8.926 mm	1.274	7.3 mm	11.5 mm	0.114
	Total	46	8.606 mm	1.353	6.1 mm	11.5 mm	-
Depth	U/L cleft	23	7.643 mm	1.033	5.8 mm	9.7 mm	-
	Class III	23	6.883 mm	1.222	3.5 mm	9 mm	0.027
	Total	46	7.269 mm	1.170	3.5 mm	9.7 mm	-
Diameter	U/L cleft	23	11.170 mm	1.840	7.3 mm	13.7 mm	-
	Class III	23	10.283 mm	0.793	8.8 mm	11.9 mm	0.039
	Total	46	10.736 mm	1.453	7.3 mm	13.7 mm	-

Shape of the sella turcica

An abnormal shape of the sella was found in both class III and cleft cases, but normal sella was found in more class III patients when compared to cleft patients. The normal morphology accounted for only 13.04% of all the cleft patients, whereas 39.13% of the class III cases exhibited a normal shape of the sella (Table 2).

**Table 2:** Frequency of the morphology of the sella This table depicts the frequency of the morphology of the sella in different groups

Sella shape	Unilateral cleft group		Skeletal class III group		Total	
	Frequency	Percent	Frequency	Percent	Frequency	Percent
Normal	3	13.04	9	39.13	12	26.08
Oblique anterior wall	4	17.39	2	8.69	6	13.04
Double contour of the floor	3	13.04	1	4.34	4	8.69
Sella turcica bridge	7	30.43	5	21.73	12	26.08
Irregularity in the posterior wall of dorsum sellae	2	8.69	3	13.04	5	10.86
Pyramidal shape of dorsum sellae	4	17.39	3	13.04	7	15.21
Total	23	-	23	-	46	-

## Discussion

Although the data that establishes a baseline distribution already exists for the Indian population, no study has compared this normative data in cleft and skeletal class III patients. This retrospective study describes the shape and size of the sella turcica in skeletal class III and unilateral cleft patients.

Size of the sella turcica

In the current study, the length of the sella ranged from 7.3 to 11.5 mm in class III patients with a mean value of 8.603+1.353 mm. The data is in agreement with AL-Mohana et al., who carried out their study on the Yemeni population and concluded that the mean length in unilateral cleft patients was 8.48 mm [14]. Alkofide found the mean length of the sella to be 11.4+2.857 mm, which is contradictory to the mean length of our study [12]. The results of this study are also different from the values observed in the study by Akay et al [15]. He observed that the mean length was 10.68+0.38 mm.

The length of the sella turcica in unilateral cleft cases according to the present study ranged from 6.1 to 11 mm with a mean value of 8.287+1.411 mm. Similar results were obtained in the studies done by AL-Mohana et al. and Akay et al. [14,15]. These results are contradicting those obtained by Alkofide. The mean depth of the sella turcica in class III patients that was obtained in the present study was 6.883+1.222 mm. The depth ranged from 3.5 to 9 mm. Comparable results were obtained in the study that was done by Tetradis and Kantor, where the mean depth was found to be 8.1 mm [16]. In the current study, the mean depth of the sella turcica in cleft patients was 7.643+1.033 mm. Yasa et al. concluded that the mean depth of the sella in cleft patients was 7.735 mm, which is similar to the results obtained in the present study [17]. In the present study, the diameter in class III cases ranged from 8.8 to 11.9 mm with a mean value of 10.283+0.793 mm, whereas in cleft cases, it ranged from 7.3 to 13.7 mm with a mean value of 11.170+1.840 mm. According to Nagaraj et al. (2015), the anteroposterior diameter of the sella ranged from 4.32 to 17.23 mm with a mean value of 11.83+1.847 mm [18].

Shape of the sella turcica

Gordon and Bell (1925) were the first to classify the morphological characteristics of the sella turcica. They categorized it into three groups: oval, circular, and saucer-shaped [19]. They found that the oval and circular shapes were most frequently present in normal subjects. This study lead to the dissemination of the morphometric analysis of the sella turcica. This motivated many researchers to conduct studies on this aspect. In 2005, Jones et al. [20] studied the morphological appearance of the sella turcica in orthodontically treated patients and found results that were similar to Gordon and Bell [19].

Because of the difficulty faced in classifying some cases into one of the three categories, Axelsson et al. (2004) classified the morphology of the sella into the six types that have been used in the current study [9]. Axelsson et al. found that two-third of the subjects had a normal sella. However, a clear diagnosis cannot be done only on the basis of the shape of the sella turcica because normal morphology was found in both normal and patients with craniofacial deformities. In the present study, 39.13% of class III patients presented with normal sella. Similar results were recorded by Chauhan et al., who found normal sella in 28% of cases [21]. This is contradictory to the study carried out by Axelsson et al. [9] in the year 2004, who concluded with his study that 66% of the Norwegian population had normal sella, whereas Silveira et al. [22] concluded that 88.4% of Brazilian people had normal morphology of the sella. This great variability in the results regarding the morphology of the sella may be due to ethnic variability.

Although it is not uncommon for the sella turcica bridge to be present in normal patients, its incidence increases in patients with craniofacial deviations. Already existing studies suggest that the sella turcica bridge may be present in 5%-22% of the population [19-22]. This study shows similar results, with 21.73% of class III patients and 30.43% of cleft patients showing the presence of a bridge. Similar to the study by Alkofide, this study also suggests an increased number of abnormal appearances of the sella turcica in cleft cases with 86.96% of the subjects having the morphology of the sella that was other than normal [12]. This is as opposed to only 60.87% of class III patients exhibiting deviation from the normal shape of the sella.

## Conclusions

The sella turcica is one of the most important landmarks used in cephalometric analysis. There are six main morphological shapes of a sella as seen in the lateral cephalogram. The most frequently seen shape of the sella in the skeletal class III group was normal shape of 39.13%, whereas the most common shape for the cleft group was the sella turcica bridge with a frequency of 30.43%. The dimensional analysis of the sella can be done using three measurements. There is no difference in the length of the sella when compared within the two groups, but there is a difference in the depth and the diameter. These results can serve as reference data for future studies.
